# Clinical Disorders in a Post War British Cohort Reaching Retirement: Evidence from the First National Birth Cohort Study

**DOI:** 10.1371/journal.pone.0044857

**Published:** 2012-09-19

**Authors:** Mary B. Pierce, Richard J. Silverwood, Dorothea Nitsch, Judith E. Adams, Alison M. Stephen, Wing Nip, Peter Macfarlane, Andrew Wong, Marcus Richards, Rebecca Hardy, Diana Kuh, on behalf of the NSHD Scientific and Data Collection Teams

**Affiliations:** 1 MRC Unit for Lifelong Health & Ageing, London, England; 2 Department of Non-Communicable Disease Epidemiology, London School of Hygiene and Tropical Medicine, London, England; 3 Manchester Academic Health Science Centre, University of Manchester, Manchester, England; 4 MRC Human Nutrition Research, Elsie Widdowson Laboratory, Cambridge, England; 5 Electrocardiology Section, Royal Infirmary, University of Glasgow, Glasgow, Scotland; Cardiff University, United Kingdom

## Abstract

**Background:**

The medical needs of older people are growing because the proportion of the older population is increasing and disease boundaries are widening. This study describes the distribution and clustering of 15 common clinical disorders requiring medical treatment or supervision in a representative British cohort approaching retirement, and how health tracked across adulthood.

**Methods and Findings:**

The data come from a cohort of 2661 men and women, 84% of the target sample, followed since birth in England, Scotland and Wales in 1946, and assessed at 60–64 years for: cardio and cerebro-vascular disease, hypertension, raised cholesterol, renal impairment, diabetes, obesity, hypothyroidism, hyperthyroidism, anaemia, respiratory disease, liver disease, psychiatric problems, cancers, atrial fibrillation on ECG and osteoporosis. We calculated the proportions disorder-free, with one or more disorders, and the level of undiagnosed disorders; and how these disorders cluster into latent classes and relate to health assessed at 36 years. Participants had, on average, two disorders (range 0–9); only 15% were disorder-free. The commonest disorders were hypertension (54.3%, 95% CI 51.8%–56.7%), obesity (31.1%, 28.8%–33.5%), raised cholesterol (25.6%, 23.1–28.26%), and diabetes or impaired fasting glucose (25.0%, 22.6–27.5%). A cluster of one in five individuals had a high probability of cardio-metabolic disorders and were twice as likely than others to have been in the poorest health at 36 years. The main limitations are that the native born sample is entirely white, and a combination of clinical assessments and self reports were used.

**Conclusions:**

Most British people reaching retirement already have clinical disorders requiring medical supervision. Widening disease definitions and the move from a disease-based to a risk-based medical model will increase pressure on health services. The promotion of healthy ageing should start earlier in life and consider the individual's ability to adapt to and self manage changes in health.

## Introduction

There is a longstanding and ongoing debate about the widening boundaries of disease and the costs and benefits of the medicalisation of health [1]–[5]. This debate is particularly relevant for the very old where multiple morbidity and polypharmacy are common and complicate treatment planning [6], and where medical problems may be overlooked or ignored [2]. It is also relevant for the early detection of disease at younger ages; for example, recent recommendations to screen everyone over 45 years for risk factors for cardiovascular disease [7] or prescribe statins or some form of ‘polypill’ for all those over 55 years old [8] would increase the proportion of the population with a disorder label and requiring regular medical supervision.

This debate needs to be informed about the current health status and treatment of older people. For the oldest old, in England, the Newcastle 85+ cohort [9] provides a description of their health status and treatment, and shows that nobody in this sample was disease free. National health surveys [10]–[12] also provide relevant information about the older population but they typically focus on different aspects of health in different years; and because they consist of a different population sample each year, it is impossible to estimate the extent of multiple morbidity. Moreover there is no way of defining in these surveys who is ‘well’ in the sense of being disease free.

In a national sample of the British population reaching retirement age, we investigate the extent of common clinical disorders, defined as an impairment of body system or structure for which there is evidence or a consensus for medical intervention, in terms of active monitoring or treatment. The data come from the Medical Research Council (MRC) National Survey of Health and Development (NSHD), a birth cohort study of men and women born in 1946 and followed ever since [13], [14]. The generation that this cohort represents is important to study, being the first of the post World War II baby boom, and benefitting from increased longevity compared with earlier cohorts. This generation will contribute to the growing proportion of the population aged 65 years and over, which is expected to rise in the UK by 32% from 11.8 million in 2008 to reach 15.6 million by 2033 [15]. The health of the baby boomers as they age will dominate the work of the health and social care systems for the next three decades, not only in the UK but in all Western developed nations.

In this cohort aged 60–64 years, we describe: the proportions with, and sex differences in, fifteen common clinical disorders of later life; the extent to which these clinical disorders are already diagnosed and/or treated; how these disorders are distributed and relate to current self reported health; and how these disorders cluster into latent classes and relate to health status previously assessed at age 36 years.

## Methods

### Ethics statement

The study protocol received ethical approval from the Central Manchester Research Ethics Committee for data collection taking place in Manchester, Birmingham, Cardiff and London. Ethical permission was given by the Scotland A Research Ethics Committee for the data collection taking place in Edinburgh. Written informed consent was obtained from the study member at each stage of data collection.

### Participants

The MRC NSHD is a socially stratified sample of 5362 singleton children born in one week in March 1946 in England, Scotland and Wales [14]. From an initial maternity survey in 1946 [16], the sample consisted of all single births to married women with husbands in non-manual and agricultural employment and one in four of all comparable births to women with husbands in manual employment. Of the 5362 original study members, the study team was still in contact with 3163 (59%) at age 60–64 years; 718 (13.4%) had died, 594 (11.1%) had previously withdrawn from the study, 567 (10.6%) lived abroad and 320 (5.9%) had been untraceable for more than ten years.

Study members received postal questionnaires between 2007 and 2008 and were invited for clinic visits between October 2007 and February 2011. If study members were unable or unwilling to come to one of the six Clinical Research Facilities (Edinburgh, Manchester, Cardiff, Birmingham, UCLH London or St Thomas' London) they were offered a slightly less comprehensive examination carried out in their own home by a trained nurse.

Of the 3163 people in the target sample, information was obtained from the postal questionnaire and/or visits from 2661 (84%). Of these, 2462 people completed the postal questionnaire and 2229 had a visit, of whom 1690 attended a clinic and 539 had a home visit.

### Self-reported disorders

For stroke, diabetes, cancer, angina, myocardial infarction (MI) and thyroid disease we went back to the first occasion on which participants were asked if they had ever had a doctor diagnosis of these conditions. These reports were updated at subsequent data collections. In 1989, participants were first asked if they had ever been told by a doctor that they had had a stroke, diabetes or cancer; in 1999 a similar question referred to angina, MI and thyroid disease; and in the most recent data collection questions on coronary artery bypass graft, angioplasty or stent were included. Cancers were also picked up from registrations on the NHS Information Centre Registry.

### Symptom scales

At age 60–64 years participants completed postal versions of the MRC Chronic Bronchitis [17] and the Rose Angina and Intermittent Claudication [18] questionnaires. The General Health Questionnaire (GHQ-28) [19] which asks about affective symptoms was self completed during the clinic or home visit.

### Prescribed medications

As part of the postal questionnaire, participants were asked to list all their prescribed medications, and the reasons why these were prescribed. These were checked by the nurses and used to help define some disorders (see below).

### Clinical assessments

Full details of the research protocol can be found elsewhere [14] but brief descriptions are given below. Those visited by a nurse at home received the same assessment except for the scans.

#### Cardiovascular assessment

Two measures of systolic and diastolic blood pressure were taken with an OMROM 705 with the participant seated, with the second reading used in this analysis. Participants visiting the clinic also had a 12 lead electrocardiogram recorded (Burdick Eclipse 850i). The output was sent by modem to the ECG Core Laboratory at the University of Glasgow for reporting.

#### DXA bone scans

Bone density scans were performed in the lumbar spine (L1–4) and proximal femur (femoral neck, total hip) using Hologic QDR 4500 Discovery scanners (Hologic Inc., Bedford MA, USA) according to standard protocols developed by Prof. J. Adams. Scans were sent to the central core laboratory in the University of Manchester for analysis.

#### Blood sample

Participants were asked to fast from 22.00 hours on the night before the visit, and the majority of blood samples were collected between 08.00 and 09.00 hours the following day. Samples were processed on site according to standardised protocols. Full blood counts were undertaken at the local hospital on the day of the visit. Aliquots were dispatched to Addenbrooke's Hospital Cambridge for HbA1c to be measured.

The remaining aliquots were stored at −80°C and couriered monthly, on dry ice, to the MRC Human Nutrition Research (HNR) laboratory in Cambridge. Serum lipids, fasting blood glucose, liver function tests, and serum creatinine were measured at HNR, and thyroid function tests at Addenbrooke's Hospital.

Serum total cholesterol, HDL cholesterol, triglycerides, glucose, and all liver function test parameters were measured colorimetrically on a Siemens Dimension Xpand analyser. LDL cholesterol was calculated from total cholesterol. HbA1c was analysed using the TOSOH G7 HPLC system. TSH and Free T4 were analysed using the Siemens Centaur Automated Immunoassay System. Free T3 was analysed using the Perkin Elmer AutoDELFIA Automated Immunoassay System until June 2009 and thereafter using the Siemens Centaur. Serum creatinine measurements were carried out using the Jaffe method.

### Defining the clinical disorders

We assessed 15 clinical disorders expected in people of this age group: cardio/cerebro-vascular disease, hypertension, raised cholesterol, renal impairment, diabetes, obesity, hypothyroidism, hyperthyroidism, anaemia, respiratory disease, liver disease, psychiatric problems, cancers, ECG abnormalities, and osteoporosis.

Clinical disorder status was derived from one or more of: a self–report of a doctor diagnosis of disease in response to a specific question; being positive on a validated scale for the disorder; a clinical assessment; currently receiving prescribed treatment for the disorder; or cancer registrations. The data sources and definitions for each clinical disorder are given in Table 1 [17]–[27]. For some conditions (hypertension, raised cholesterol, renal impairment, diabetes, obesity, hypothyroidism, and hyperthyroidism) two levels of disorder were defined, level 1 where intervention is required irrespective of other information and level 2 where the type of intervention may depend on other information. For example, whether or not to treat a person with a raised cholesterol level depends on the overall 10 year risk of a significant event.

**Table 1:** Clinical disorders, level 2 and level 1, with data sources and definitions.

Clinical disorder	Level 2/level 1	Constituent sub-disorders/definition
Level 1 CVD	Level 1	i) Angina (self-report of doctor diagnosed angina or self-reported by Rose chest pain questionnaire [18];ii) CABG (self-report of CABG);iii) Angioplasty/stent (self-report of angioplasty or stent);iv) MI (self-report of doctor diagnosed MI);v) Stroke (self-report of doctor diagnosed stroke); orvi) Intermittent claudication (self-reported by Rose intermittent claudication questionnaire [18].
Hypertension	Level 1	Current SBP≥160 mmHg or current DBP≥100 mmHg, or reported being on anti-hypertensive medication.
	Level 2	Current SBP≥140 mmHg or current DBP≥90 mmHg but were not included in the ‘level 1 hypertension’ group.
Raised cholesterol	Level 1	Total∶HDL cholesterol ratio ≥6.0 mmol/l or reported being on statins or fibrates.
	Level 2	Chance of developing a cardiovascular disease within the next 10 years≥20% according to the Framingham risk score [20] but not included in the ‘level 1 raised cholesterol’ group. Risk score was calculated using current age, LDL and HDL cholesterol, current SBP and DBP, current smoking status (self-reported by direct questions), and ‘level 1 diabetes’ (see below).
Renal impairment	Level 1	eGFR<30 ml/min/1.73 m^2^ [21]. eGFR calculated from serum creatinine using the MDRD formula [22].
	Level 2	30≤eGFR<60 ml/min/1.73 m^2^ or dipstick proteinuria≥+ [22].
Diabetes	Level 1	FBG≥7 mmol/l (restricted to study members who reported fasting appropriately), self-report of doctor diagnosed diabetes, or reported being on diabetes medication.
	Level 2	6.0>FBG<7 mmol/l (restricted to study members who reported fasting appropriately) [23].
Obesity	Level 1	BMI≥40 kg/m^2^ [20]. BMI calculated as weight (m)/height (kg)^2^ [24].
	Level 2	30 kg/m^2^≤BMI]<40 kg/m^2^ [24].
Hypothyroidism	Level 1	Self-report of doctor diagnosed hypothyroidism, TSH>5.5 mU/l and free T4<10.0 pmol/l (modified from Wilson 2006 [25]), or reported being on thyroxine.
	Level 2	TSH>5.5 mU/l and 10.0≤free T4≤19.8 pmol/l (modified from Wilson 2006 [25]).
Hyperthyroidism	Level 1	Self-report of doctor diagnosed hyperthyroidism, TSH<0.4 mU/l and free T3>7.5 pmol/l (modified from Wilson 2006 [25]), or reported being on medication.
	Level 2	TSH<0.4 mU/l and 3.0≤free T3≤7.5 pmol/l and 10.0≤free T4≤19.8 pmol/l (modified from Wilson 2006 [25]).
Anemia	Level 1	Haemoglobin <13 g/dl (males) or haemoglobin <12 g/dl (females) [26].
Respiratory disease	Level 1	Chronic bronchitis symptoms (MRC chronic bronchitis questionnaire [17]) or reported being on medication.
Liver disease	Level 1	Albumin <25 g/l, bilirubin >100 µmol/l, alkaline phosphatase >300 IU/l, alanine transaminase >300 IU/l, aspartate transaminase >300 IU/l, or gamma glutamyl transferase >100 IU/l.
Psychiatric problems	Level 1	GHQ ‘caseness’ (≥5 items ‘worse than usual’ or ‘much worse than usual’) [19].
Cancers	Level 1	Self-report of doctor diagnosed cancer or cancer reported to the cancer registry.
ECG abnormalities	Level 1	Atrial fibrillation, atrial flutter or definite MI of any age.
Osteoporosis	Level 1	Bone density t-score by DXA scan ≤2.5 at spine, femoral neck, or hip [27].

CVD, cardiovascular disease; CABG, coronary artery bypass graft; MI, myocardial infarction; SBP, systolic blood pressure; DBP, diastolic blood pressure; HDL, high-density lipoprotein; LDL, low-density lipoprotein; eGFR, estimated glomerular filtration rate; MDRD, modification of diet in renal impairment; FBG, fasting blood glucose; TSH, thyrotrophin-stimulating hormone; T4, thyroxine; T3, triiodothyronine; MRC, Medical Research Council; GHQ, General Health Questionnaire; ECG, electrocardiogram; DXA, dual-emission X-ray absorptiometry.

### Covariates

#### Health status at 36 years

Cohort members had previously been identified as being in the best of health (10%), intermediate (65%) or worst of health (25%) at age 36 years on the basis of measured blood pressure, lung function and body weight, self reported health problems and disability, and recent hospital admission [28].

#### Self-reported health status at 60–64 years

At age 60–64 years participants were asked to rate their current health on a 5 point scale from excellent to poor.

### Statistical methods

The proportion with each clinical disorder was calculated as the number of participants meeting the relevant definition (see Table 1) divided by the number of participants with valid data for that measure. Where more than one measure was used in the definition of a disorder (for example total∶HDL cholesterol ratio and reported statin/fibrate use for level 1 raised cholesterol), the sample was restricted to those with valid data for all relevant measures. The proportions were weighted to account for the social stratification in the original sample. Unweighted proportions were also calculated for comparison.

#### Undiagnosed disease

The number of participants who had diabetes, osteoporosis, hypertension, and thyroid disorders on examination, but who had not reported a doctor diagnosis of that condition or who were not on treatment for that specific disorder was divided by the total number of participants, to give the proportion of the total sample with undiagnosed disease. The proportion of those undiagnosed within the sample who had each of these disorders was also calculated. Social class-weighted results are presented, but unweighted results were produced for comparison.

#### Distribution and clustering of clinical disorders

For each participant for whom status on all the 15 disorders was defined, the number of identified clinical disorders was counted; this was initially restricted to level 1 disorders, then repeated to include those with level 2 disorders also. The number of disorders per participant was compared between sexes, and by self-rated health using a chi-squared test, grouping those with five or more clinical disorders together. This analysis was then repeated excluding the two disorders (osteoporosis and ECG abnormalities) that depended on the clinic visit for ascertainment. We assessed whether the number of disorders varied according to whether participants had a clinic or home visit, and by current self reported health status. Both weighted and unweighted analyses were again conducted.

To explore further the clustering of level 1 clinical disorders, latent class analysis (LCA) was performed on the level 1 clinical disorder variables. Latent class analysis is a multivariable regression model that describes the relationships between a set of observed dependent variables (‘latent class indicators’), in this case the clinical disorders, and an unobserved categorical latent variable, each level of which is referred to as a ‘latent class’. For dichotomous latent class indicators, as in the present application, the relationships are described by a set of logistic regression equations [29]. LCA assumes conditional independence of variables within each latent class. The objective is to identify the latent class indicators that best distinguish between classes and to categorise people into their most likely classes given their observed responses [30]. We used a variety of different tools to decide how many classes were required as no single approach is commonly accepted: the Lo-Mendell-Rubin adjusted likelihood ratio test (LRT), the bootstrap LRT, and three information criteria – Akaike's Information Criterion (AIC), Schwarz's Bayesian Information Criterion (BIC), and sample size-adjusted BIC (aBIC). The entropy, relative sizes, and meaningful interpretation of the latent classes were also considered. LCA was performed on complete cases only, then repeated using all study members who contributed data on at least one clinical disorder using full information maximum likelihood (FIML) under the assumption of missing at random for comparison [31]. LCA was conducted using Mplus version 6 [32].

The relationship between the latent classes and health status at 36 years was investigated using logistic regression of each latent variable on health status, weighted by the LCA posterior class membership probabilities.

## Results

2657 survey members contributed to the analysis for one or more clinical disorders. Of these, 1104 (41.6%) belonged to the manual social class strata as defined at the initial sampling in 1946. This compares to 2370 (44.2%) of the 5362 study members originally in the cohort.

The commonest disorders were level 1 or level 2 hypertension (54%), obesity (31%), raised cholesterol (27%), and diabetes or impaired fasting glucose (26%). Other disorders affecting at least one in ten people were psychiatric disorders (19%), chronic respiratory disease (12%), cancers (11%), osteoporosis (11%), cardiovascular disease (11%) and renal impairment (10%); hypothyroidism (7%) and liver disease (5%) were less common. There was strong evidence for sex differences in many of the conditions examined. The others showed weak evidence (intermittent claudication, level 2 hyperthyroidism and cancers) or no evidence (anaemia, chronic respiratory disease, level 2 obesity and level 2 renal impairment); the number with level 1 renal impairment was too low to detect a sex difference. Hypertension, raised cholesterol, diabetes, cardiovascular problems, ECG abnormalities and abnormal liver function were more common in men, and psychiatric problems, thyroid disease, osteoporosis, and level 1 i.e. morbid obesity were more common in women (Table 2). The unweighted prevalences were similar for most clinical disorders, but slightly lower for several (results not shown). This would be expected if the disorders were more prevalent in the manual social class, of which a smaller fraction were sampled.

**Table 2:** Number and percent with level 1 and level 2 clinical disorders in men and women 60–64 years in the MRC National Survey of Health and Development.

		Males		Females		Total		
Clinical disorder	Level 2/level 1	n/N	% (95% CI)^A^	n/N	% (95% CI)^A^	n/N	% (95% CI)^A^	P for diff between sexes
Level 1 CVD								
Angina	Level 1	97/1164	9.2 (7.3, 11.4)	60/1263	5.2 (3.9, 6.9)	157/2427	7.1 (5.9, 8.5)	0.002
CABG	Level 1	22/1136	2.3 (1.4, 3.6)	3/1209	0.3 (0.1, 1.2)	25/2345	1.3 (0.8, 2.0)	0.001
Angioplasty/stent	Level 1	40/1127	3.7 (2.6, 5.3)	13/1205	1.3 (0.7, 2.4)	53/2332	2.5 (1.8, 3.4)	0.002
MI	Level 1	59/1146	5.8 (4.3, 7.7)	15/1215	1.7 (0.9, 2.9)	74/2361	3.7 (2.8, 4.7)	<0.001
Stroke	Level 1	34/1181	3.1 (2.1, 4.6)	21/1268	2.1 (1.3, 3.4)	55/2449	2.6 (1.9, 3.5)	0.21
Intermittent claudication	Level 1	13/1152	1.4 (0.8, 2.6)	6/1235	0.4 (0.2, 1.1)	19/2387	0.9 (0.5, 1.5)	0.03
Any level 1 CVD	Level 1	133/1048	13.8 (11.5, 16.6)	71/1093	7.5 (5.8, 9.7)	204/2141	10.6 (9.1, 12.3)	<0.001
Hypertension	Level 1	407/1061	41.2 (37.6, 44.8)	348/1147	33.0 (29.8, 36.3)	755/2208	36.8 (34.4, 39.3)	0.001
	Level 2	225/1061	20.2 (17.5, 23.2)	188/1147	14.9 (12.6, 17.5)	413/2208	17.4 (15.6, 19.4)	0.005
Raised cholesterol	Level 1	269/960	28.0 (24.7, 31.5)	176/1024	18.2 (15.5, 21.3)	445/1984	22.9 (20.7, 25.2)	<0.001
	Level 2	63/785	8.9 (6.7, 11.6)	5/838	0.4 (0.1, 1.3)	68/1623	4.5 (3.4, 5.9)	<0.001
Renal impairment	Level 1	2/913	0.2 (0.0, 1.2)	0/942	0.0 (NA, NA)	2/1855	0.1 (0.0, 0.6)	NA
	Level 2	95/895	11.4 (9.1, 14.2)	78/932	9.4 (7.3, 12.0)	173/1827	10.4 (8.8, 12.3)	0.25
Diabetes	Level 1	101/834	12.8 (10.3, 15.9)	66/907	7.4 (5.6, 9.7)	167/1741	9.9 (8.4, 11.8)	0.002
	Level 2	180/917	20.5 (17.5, 23.9)	113/982	11.9 (9.7, 14.6)	293/1899	16.0 (14.1, 18.1)	<0.001
Obesity	Level 1	10/1061	0.7 (0.3, 1.5)	31/1158	3.0 (2.0, 4.5)	41/2219	1.9 (1.3, 2.7)	<0.001
	Level 2	290/1061	29.3 (26.1, 32.7)	320/1158	29.1 (26.0, 32.3)	610/2219	29.2 (26.9, 31.5)	0.93
Hypothyroidism	Level 1	20/903	1.8 (1.0, 3.0)	107/969	10.9 (8.7, 13.5)	127/1872	6.5 (5.3, 8.0)	<0.001
	Level 2	18/1000	1.5 (0.9, 2.7)	67/1063	6.4 (4.8,8.4)	85/2063	4.1 (3.2, 5.2)	<0.001
Hyperthyroidism	Level 1	6/903	0.6 (0.2, 1.6)	22/968	2.7 (1.7, 4.3)	28/1871	1.7 (1.1, 2.6)	0.003
	Level 2	7/1000	0.6 (0.2, 1.5)	17/1062	1.5 (0.8, 2.6)	24/2062	1.0 (0.6, 1.7)	0.09
Anemia	Level 1	45/999	3.8 (2.7, 5.5)	48/1063	5.1 (3.7, 7.0)	93/2062	4.5 (3.6, 5.7)	0.23
Respiratory disease	Level 1	140/1132	12.6 (10.4, 15.1)	140/1223	12.2 (10.2, 14.6)	280/2355	12.4 (10.9, 14.1)	0.84
Liver disease	Level 1	74/1002	7.3 (5.6, 9.5)	37/1059	3.3 (2.2, 4.8)	111/2061	5.2 (4.2, 6.5)	0.001
Psychiatric problems	Level 1	137/1047	13.5 (11.2, 16.2)	250/1136	23.3 (20.4, 26.4)	387/2183	18.7 (16.8, 20.7)	<0.001
Cancers	Level 1	108/1169	9.6 (7.8, 11.9)	164/1270	12.5 (10.5, 14.8)	272/2439	11.1 (9.7, 12.7)	0.06
ECG abnormalities	Level 1	47/785	6.2 (4.5, 8.6)	14/846	2.1 (1.1, 3.7)	61^B^/1631	4.1 (3.0, 5.4)	0.001
Osteoporosis	Level 1	50/780	7.3 (5.3, 10.0)	129/853	14.0 (11.5, 17.0)	179/1633	10.9 (9.2, 12.8)	<0.001

A Weighted according to original social class-stratified sampling. CVD, cardiovascular disease; CABG, coronary artery bypass graft; IC, intermittent claudication; MI, myocardial infarction; ECG, electrocardiogram.

B Of whom 31(2.3%) had atrial fibrillation.

### Previously undiagnosed disorders

Table 3 shows the proportions with undiagnosed hypertension, diabetes, thyroid disease, and osteoporosis in the responding sample. Nine percent of the sample had undiagnosed untreated osteoporosis (79% of those with osteoporosis), 6% undiagnosed hypertension (15% of those with hypertension), 3% undiagnosed diabetes (39% of those with diabetes), and <1% undetected thyroid disease (8% of those with thyroid disease). Undiagnosed hypertension and diabetes were commoner in men. Undiagnosed osteoporosis was commoner in women in the full sample; however, within the group with osteoporosis, men were more likely to be undiagnosed (90%) than women (74%). There were no evidence of sex differences in undiagnosed thyroid disorders. The results from the unweighted analysis were very similar to those in the weighted analysis (results not shown).

**Table 3:** Diagnosed and undiagnosed level 1 clinical disorders.

	n/N (%^A^)						
	Males		Females		Total		
Clinical disorder	Diagnosed	Undiagnosed	Diagnosed	Undiagnosed	Diagnosed	Undiagnosed	P for diff in undiagnosed between sexes
Hypertension	331/1061 (32.5)	76/1061 (8.7)	309/1147 (29.5)	39/1147 (3.5)	640/2208 (30.9)	115/2208 (5.9)	<0.001
Diabetes	58/834 (8.0)	43/834 (4.8)	43/907 (5.2)	23/907 (2.2)	101/1741 (6.5)	66/1741 (3.4)	0.01
Hypothyroidism	19/903 (1.6)	1/903 (0.2)	99/969 (10.2)	8/969 (0.6)	118/1872 (6.1)	9/1872 (0.4)	0.26
Hyperthyriodism	5/903 (0.6)	1/903 (0.1)	19/968 (2.4)	3/968 (0.3)	24/1871 (1.5)	4/1871 (0.2)	0.12
Osteoporosis	5/780 (0.5)	45/780 (6.8)	33/853 (3.7)	96/853 (10.3)	38/1633 (2.2)	141/1633 (8.6)	0.04

A Weighted according to original social class-stratified sampling.

### Distribution of clinical disorders and relationship to current self reported health

Table 4 shows the distribution for the number of level 1 clinical disorders for those with information on all 15 or 13 clinical disorders. Thirty percent of the participants were free of all of the 15 level 1 disorders. The results on the larger sample, excluding the conditions only defined at the clinic visit (osteoporosis and ECG abnormalities), were similar, but slightly more participants (33%) were disorder free. In both cases the median number of level 1 disorders was 1 (range 0–8), and there was no evidence of sex difference in the number of disorders.

**Table 4:** Number (percent) of level 1 clinical disorders per study member.

	n (%^A^)					
	All clinical disorders (max. 15)^B^			Excluding osteoporosis and ECG abnormalities (max. 13)^C^		
Number of clinical disorders	Males	Females	Total	Males	Females	Total
0	146 (26.3)	171 (32.4)	317 (29.5)	194 (30.8)	222 (34.2)	416 (32.6)
1	178 (34.4)	162 (29.6)	340 (31.8)	206 (32.0)	198 (31.6)	404 (31.8)
2	84 (18.2)	114 (19.6)	198 (18.9)	96 (16.1)	115 (17.6)	211 (16.9)
3	63 (12.8)	53 (10.5)	116 (11.6)	72 (12.5)	58 (10.0)	130 (11.2)
4	28 (5.2)	24 (4.4)	52 (4.8)	31 (5.5)	22 (3.8)	53 (4.6)
5+	14 (3.3)	15 (3.6)	29 (3.5)	16 (3.0)	13 (2.7)	29 (2.8)
Total	513	539	1052	615	628	1243

A Weighted according to original social class-stratified sampling.

B Range: 0–8; median: 1; *P* value for diff between sexes = 0.46.

C Range: 0–8; median: 1; *P* value for diff between sexes = 0.60.

Table 5 shows the number of level 1 and level 2 clinical disorders in those with information on all 15 or 13 clinical disorders. Only 15% were free of either a level 1 or level 2 disorders when 15 clinical disorders were considered, and 16% were disorder free when 13 clinical disorders were considered. In both cases the median number of level 2 or level 1 disorders was 2 (range 0 to 9). Women were somewhat more likely to be disorder free.

**Table 5:** Number (percent) of level 1-level 2 clinical disorders per study member.

	n (%^A^)					
	All clinical disorders (max. 15)^B^			Excluding osteoporosis and ECG abnormalities (max. 13)^C^		
Number of clinical disorders	Males	Females	Total	Males	Females	Total
0	69 (11.3)	92 (17.7)	161 (14.7)	91 (13.8)	117 (18.2)	208 (16.1)
1	116 (22.5)	128 (23.4)	244 (23.0)	148 (23.9)	160 (26.7)	308 (25.3)
2	108 (24.1)	128 (24.8)	236 (24.4)	119 (21.8)	150 (24.8)	269 (23.3)
3	89 (18.0)	83 (16.4)	172 (17.2)	103 (17.1)	90 (15.5)	193 (16.3)
4	61 (12.6)	50 (9.6)	111 (11.0)	64 (12.1)	52 (8.0)	116 (10.0)
5+	50 (11.5)	39 (8.1)	89 (9.7)	60 (11.3)	35 (6.8)	95 (9.0)
Total	493	520	1013	585	604	1199

A Weighted according to original social class-stratified sampling.

B Range: 0–9; median: 2; *P* value for diff between sexes = 0.13.

C Range: 0–9; median: 2; *P* value for diff between sexes = 0.03.

The distributions of the number of clinical disorders per study member were broadly similar in the unweighted analysis, though a slightly greater proportion of study members were disorder-free (results not shown). Again, this would be expected if disorders were more prevalent in the manual social class.

Those who had a home visit were less likely to be free from clinical disorders than those who had a clinic visit (23.3% v. 34.0%, p = 0.02 for level 1 clinical disorders).

In the total responding sample, 13% of participants described their health as excellent, 40% as very good, 32% as good, 13% as fair, and 2% as poor. These ratings were strongly associated with the number of level 1 and level 2 clinical disorders reported (p<0.001). For example, considering all 15 clinical disorders, 77% of those with no level 1 or 2 clinical disorders rated their health as excellent or very good and 2% rated it as fair or poor; but only 31% of those with 5 or more level 1 or 2 disorders rated their health as excellent or very good and 40% rated it as fair or poor. Around 50% of study members with cardiovascular disease or morbid obesity rated their health as fair or poor. These figures were essentially unchanged in the unweighted analysis.

### Clustering of clinical disorders and relationship to health status at 36 years

**Figure 1:**
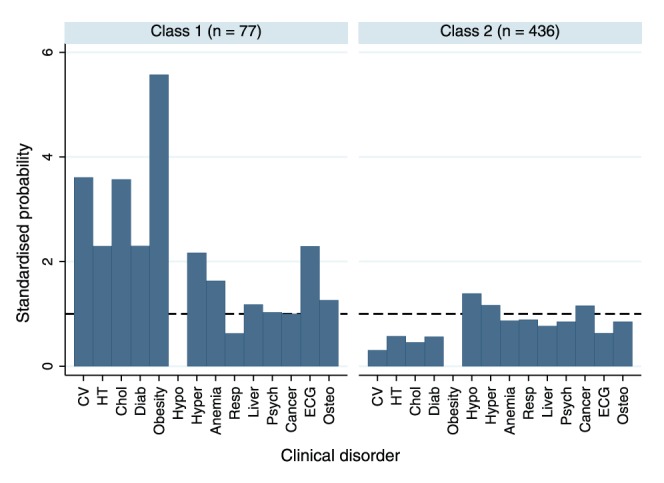
1a and 1b. Latent classes of level 1 clinical disorders (excluding renalimpairment) in males (n = 513). Standardised probabilities are calculated by dividing the probability of each clinical disorder within each latent class by the observed proportion of study members with that disorder in the sample.

**Figure 2:**
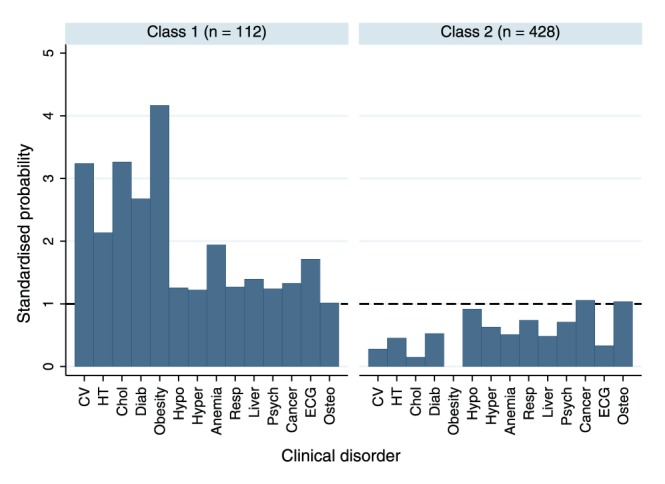
2a and 2b. Latent classes of level 1 clinical disorders (excluding renal impairment) in females (n = 540). Standardised probabilities are calculated by dividing the probability of each clinical disorder within each latent class by the observed proportion of study members with that disorder in the sample.

Level 1 renal impairment was excluded from the LCA as the prevalence was too low. The remaining 14 clinical disorders meant that there were 16384 possible patterns of clinical disorders within the dataset. There was strong evidence that LCA models in which parameters were allowed to differ between males and females provided a better fit to the data than a model which combined both sexes. For both males and females a two class model provided the best fit to the data. Figures 1 and 2 show the latent classes of clinical disorders for males and females respectively using standardised probabilities, which are calculated by dividing the probability of each clinical disorder in each latent class by the observed proportion of study members with that disorder in the sample. For both males and females there was a smaller latent class (n = 77 for men and n = 112 for women) in which participants had a high probability of cardio-metabolic disorders (Figure 1a and 2a). The remaining larger latent class for men (n = 436) and women (n = 428) had a generally low probability of disorders (Figure 1b and 2b). As this larger cluster included participants with no disorders, the analysis was repeated excluding them. This gave essentially the same result, distinguishing those with a high probability of cardio-metabolic disorders from others. Repeating the LCA using all study members who contributed data on at least one clinical disorder using FIML increased the number of study members contributing to the analysis to 1283 men and 1374 women. Two latent classes were again required for both men and women, and the probabilities of each clinical disorder within each latent class were very similar to those in the complete case analysis (results not shown). However, the classes were not as clearly separated (lower entropy) due to the inclusion of study members with reduced information.

In both males and females there was evidence of a trend in the relationship between the latent classes at 60–64 years and health status at age 36 years, with those in the worst health at age 36 more likely to be in the cardio-metabolic latent class almost thirty years later. Of those in the worst of health at 36 years, 25.5% of men and 30.9% of women were in the cardio-metabolic latent class at 60–4 years, whereas this was only true for only 13.2% of men and 14.6% of women in the best of health almost thirty years earlier (p = 0.02 and p = 0.003 respectively). When using the latent classes derived under FIML the magnitude of the associations was very similar (results not shown).

## Discussion

### Key findings

In this survey of common clinical disorders in the first wave of British baby boomers at 60–64 years, we found that most had clinical disorders even though the majority described their general health as good, or better. These disorders were widely spread across the population, with only one in six people being free of all the disorders considered i.e. not requiring any medical intervention; this distribution was strongly related to self reported health. We identified a latent class of cardio-metabolic disorders, covering one in five of the population; this class was more likely to have had poor health as young adults.

### Strengths and limitations of the study

As with all longitudinal studies NSHD has suffered from attrition. However, this is a large, national cohort that remains reasonably representative of the British born population. The NSHD study population is however all white, and therefore our findings cannot be extrapolated to the non-White British population. Non-White people make up 9% of the current population of England and Wales but they generally have a younger age profile, and only 5% of the population 60–64 years are non-White [33].

Some of our disorders are defined only on self–report of a doctor's diagnosis of disease. However, self-reports are generally found to be reasonably accurate when compared with medical records in cases of definite diagnoses [34], [35]. In the NSHD we sent questionnaires to GPs asking them to confirm self reports of chronic disease; for diabetes we found over 90% agreement [36]. We only measured blood pressure on one occasion so we may have overestimated the amount of previously undiagnosed hypertension, since in practice a diagnosis of hypertension is based on at least two readings taken at different times.

Rare disorders or common disorders in which confirmatory clinical assessments were not possible, and treatment is non-specific e.g. arthritis, were not included. Hence this study estimated the extent of common clinical disorders rather than the full extent of health problems in this cohort at retirement age.

Although our analysis of the clustering of clinical disorders within study members was presented for complete cases only, obtaining very similar results using all study members who contributed data on at least one clinical disorder suggested that significant bias was not introduced.

### Comparison of our findings with other published studies

The proportions observed for cancers, diabetes, thyroid disease, obesity and psychiatric problems were similar to those in other studies, as were those for previously undiagnosed thyroid disorders and diabetes [20], [22], [36]–[44]. The major difference between our results and other reported studies was in relation to hypertension, the NSHD population had noticeably more people with hypertension than reported in HSE 2009 [37]. Our findings on renal impairment are not directly comparable with the other UK data [37], [42] as these studies report CKD stages 3–5 (NICE 2008) [21] which exclude people with proteinuria but eGFR>60 ml/min/1.73 m^2^ from being classified as having renal impairment. In NSHD the proportion with of CKD stages 3–5 is slightly lower than for the other studies [37], [42]. However people with proteinuria alone do have a higher CVD and renal progression risk [22], and the NSHD identifies this group for the first time in a British general population. We found somewhat fewer MIs, strokes, and better respiratory function than has been reported in cross–sectional studies in people aged 64–69 in England [38], but this is probably accounted for by the age difference. NSHD women had somewhat less osteoporosis than estimated by WHO [27]; this may be an underestimation because participants who attended the clinics (where bone densitometry was available) had fewer clinical disorders than the people seen at home visits.

#### Undetected disorders

There was a high proportion of undetected osteoporosis in men and women. This is a condition that is not screened for as population screening is not cost effective [44]. National guidelines [44] do encourage case finding but this may not be widely applied and awareness of the problem in men is not high [45]. This may improve in the future with osteoporosis being introduced into the Quality Outcomes Framework in 2012. Undetected diabetes within the responding population was more frequent than found in the English Longitudinal Study of Ageing [39]; almost two fifths of those with diabetes had not been previously diagnosed. There was very little undetected thyroid disease, as found in the Birmingham Elderly Thyroid Study (BETS) [25]. This may be explained by GP awareness of thyroid disorders conditions and their success in case finding. The levels of undiagnosed hypertension and diabetes should reduce once the proposal for Vascular Screening system in the over 45 year-olds comes on-line in general practice [7].

#### Multiple disorders and clustering of disorders

In the Newcastle cohort study of those aged 85 years and older, nobody was disorder-free. This study also included heart failure, dementia and Parkinson's disease but we did not, as these conditions are rare in 65 years olds. Unlike our study, the authors found that women had more clinical disorders than men [9].

The picture we outline here is in many ways a best case scenario. It may be that that people born in the immediate post-war period are healthier than earlier or later born people because they have spent their whole lives within the post-war welfare state and only later experienced exposure to obesogenic lifestyles. There are a number of other conditions which we have not included, e.g. sarcopenia and osteopenia, that are as yet not monitored or treated but, given shifting boundaries, could easily become defined as clinical disorders [4]. We have not followed recent proposals for extending risk factors further back into the normal distribution identifying ‘prehypertension’ ( SBP≥120 mmHg ); impaired fasting glycaemia as beginning at 5.6 mmol/l ; and borderline risk of LDL as beginning at 3.6 mmol/l. In the US population Kaplan and Ong [46] estimate that such categorization of these three risk factors alone would result in 97% of the population being under medical surveillance.

The study of patterns of multi-morbidity is relatively new field. There are few studies that have examined the way conditions cluster within groups of patients. One in the USA examined the records of 1.3 million primary care patients cared for by the Veterans Health Care System with two or more co morbidities and categorized 45 health conditions [47]. They reported that 83% of their sample fell into their metabolic cluster. An Australian study in working age adults [48] found that health conditions do not cluster neatly into organ or body systems as has been assumed by methods underpinning the Cumulative Index Rating Scale [49] and they identified 6 independent clusters of disorders.

### Implications

Few people are without a clinical disorder on reaching retirement age. This highlights two sets of problems – the first is conceptual, related to our changing definition of disease and to current theories of ageing; and the second is pragmatic, and concerns the workload of health services going forward.

We have increasingly moved from a diseased-based model of medicine where doctors reacted to signs and symptoms presented by the patient, to a more proactive risk based model in which many clinical disorders are the end of a distribution of biological attributes, and are detected on the basis of case finding. This move has been driven by the success of epidemiological studies in identifying risk factors, and demonstrating effective interventions to reduce risk and to treat diseases early. Certainly this more proactive approach has coincided with a marked reduction in mortality rates for cardiovascular disease [50], although some would argue that the relationship between the two is not causal [51].

Individuals have different patterns of ageing. Such patterns have been postulated as; ‘survivors’ who live with extended morbidity due to age related disease diagnosed before old age; ‘extenders’ who live longer than expected without problems and have a shorter period of disability before death; and ‘escapers’ who attain very old age without disease [49], [50]. As life expectancy increases there is a debate about whether we are facing a compression of morbidity, with people both living longer and having a longer period of healthy life (more extenders and escapers) or an expansion of morbidity with people living longer but having little or no increase in healthy life (more survivors). Alternatively, we may be facing some intermediate state involving more people living longer but with less severe morbidity (more survivors but with less disability). It has been proposed that ageing research should focus on extenders and escapers, to identify factors related to compression of morbidity and to avoid a pandemic of disability [52], [53], and not just on age-related disease.

However, our research, and that emerging from the Netherlands [54], suggest that only a small minority of people in Western populations are ‘escapers’ or ‘extenders’, even by retirement age. Data from the Longitudinal Aging Study Amsterdam do not support the compression of morbidity scenario [54], and a study across OECD countries shows inconsistent results [55]. Thus ageing with clinical disorders may become the norm, and it might better to speak in terms of health relative to others of the same age. This situation is prompting new formulations of the meaning of health, especially in relation to older people, which focus on the individual's ability to adapt and self manage physically, psychologically and socially to their changing internal and external environment [56], [57].

With our changing definitions of disease and the ageing population comes a significant load on the health services, especially general practice, in terms of monitoring and treatment. Not only is general practice delivering its traditional reactive role but it is increasingly expected to deliver the preventive care part of the public health agenda. The effect on workload is already being seen. Over the past decade patient consultation rates per patient in England and Wales have increased over 40% (from 3.9 in 1995/6 to 5.5 per patient per year 2008/9) [58], and this is also being seen in the other OECD countries [59].

The commonest conditions in this study are cardio-metabolic, cancers and osteoporosis which share common upstream causes in the nutritional deterioration of the nation's diet and sedentary behaviour. Unless we are able to tackle successfully these upstream causes on a societal level, we will be left with treating the effects in individuals, either monitoring and trying to change behaviour or prescribing medications. Our demonstration that health status at retirement is strongly associated with health status almost thirty years earlier suggests that a high risk group could be identified that may benefit from individual level interventions that start earlier than middle age. The current structure of the clinical care is still based on the disease model, and may be overwhelmed by the demands of proactive care. The purpose of this paper is not to give solutions to these problems, but to highlight the current position, and emphasize that the proportion of the population ‘under the doctor’ will only increase given an ageing population and diagnostic creep.
